# Supplementary material for: Subjective affective experience under threat is shaped by environmental affordances
Source: PLoS One. 2024 Dec 12;19(12):e0310359. doi: 10.1371/journal.pone.0310359 (PMC11637274; doi:10.1371/journal.pone.0310359)
Supplement: S1 File — (PDF) [file pone.0310359.s001.pdf]

Supporting Information

Sample size rationale

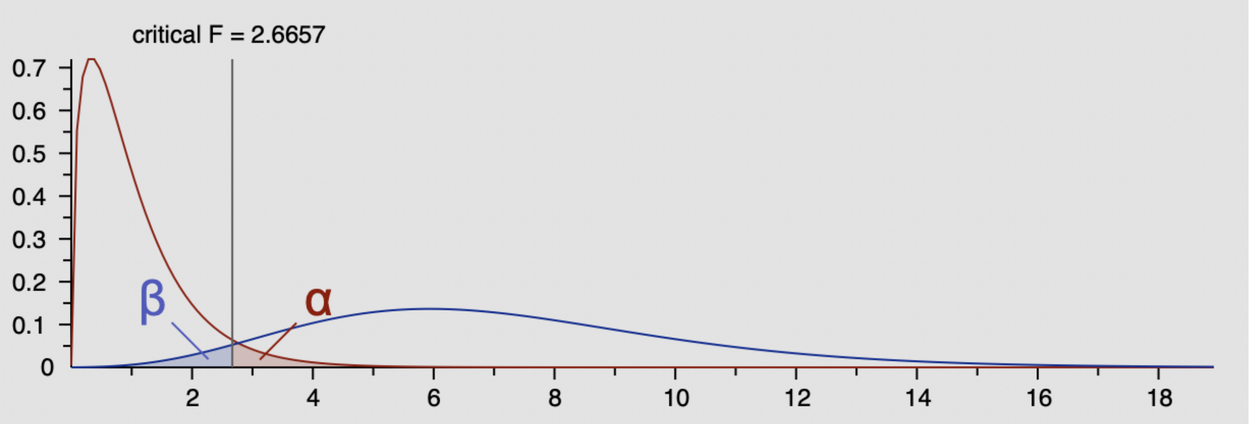

We performed the power analysis using G-power (cite). For a target effect size f = 0.25, and power = 0.95, and an assumption of correlation among repeated measures = 0.5, the estimated required sample size is **n = 152**. The simulated actual power is 0.95514.

Sample size rationale (Bayesian)
The Bayesian power is calculated from the pilot data, on the full model (*anger_rating ~ anxiety + stim_rating+ affordance + choice + affordance:choice + (affordance + choice + affordance:choice|subject) + (affordance + choice + affordance:choice|type)*), as the percentage of simulations that has a lower limit of the 95% interval (Q2.5) greater than 0, indicating a non-trivial effect. Specifically, the power is calculated for the interaction term, Affordance X Choice.

We set i = 100 as the increment of simulation indices. With n = 100, the Bayes Power = 0.488; And when **n= 250**, the Bayes Power = 0.868.

Standalone rating task

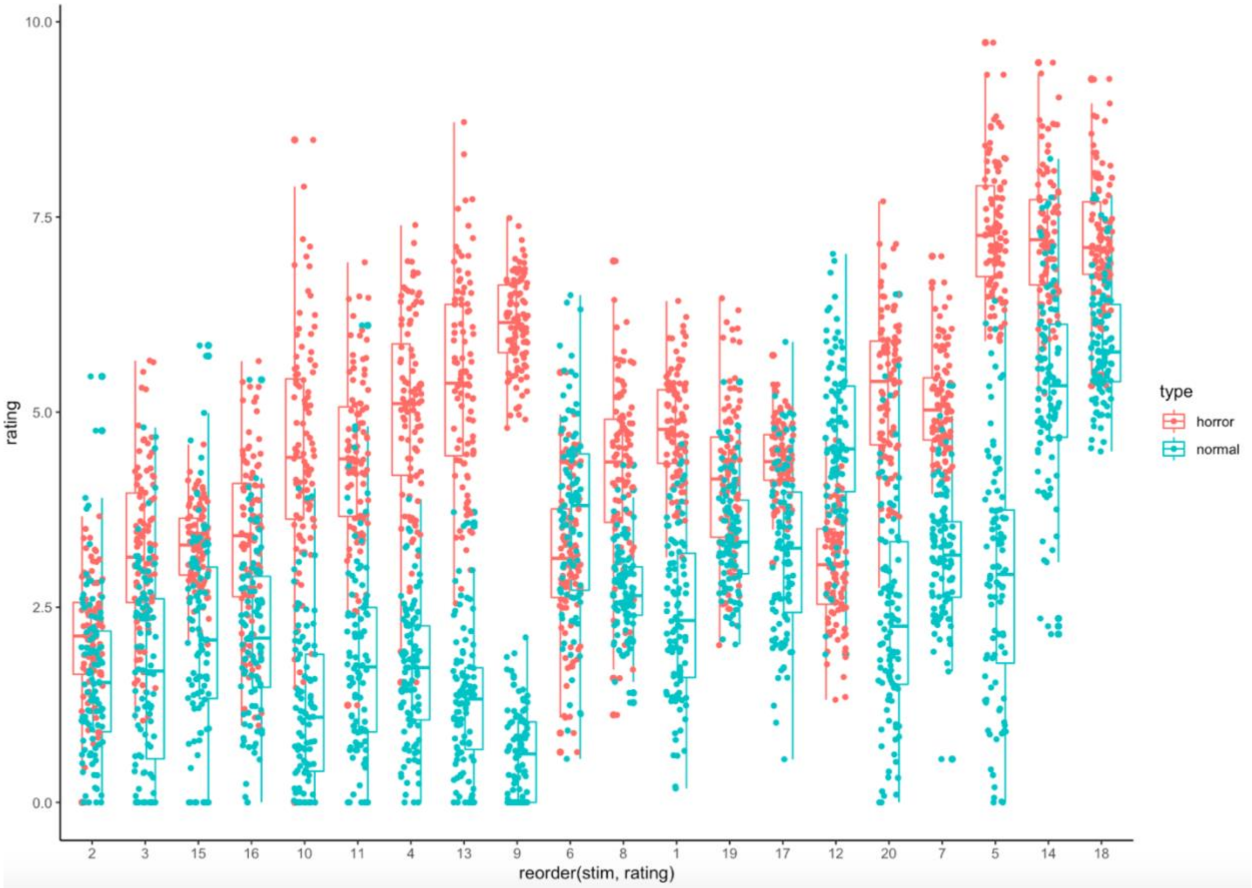

Ratings scores for neutral (green) and horror (red) clips. Note that for each stimulus pair, the neutral clips were obtained from the same movie scene as the horror clips.

In the independent rating task, a different set of Mturk participants (sample size = 100) were asked to rate the fearfulness of the horror movie clips used in the main experiment. They were only presented with the movie clips alone, without the affordances nor decisions to make. This is a proof of concept that the horror movies are scary enough to elicit defensive responses and generate adaptive behavioral choices. Overall, participants' mean rating of fearfulness was 4.49 (3.98), with a relatively balanced distribution over different stimuli.

We then compared the rating data from the horror scene clips to the control experiment, where neutral clips from the same movies were paired and used. Bearing a few exceptions, most horror scenes were rated as more scary than the neutral scenes ($t(19) = 41.03$, $p < 0.001$). This confirms that the high fearfulness ratings for the selected clips were not from the natural spectrum from the rating distribution, and that the horror movies clips were effective in inducing fear.

Bayesian Models

We constructed two Bayesian mixed effects model to look at the relationship between emotional ratings and decisions. The full models are expressed in pseudo-code as shown below:

anger_rating ~ trait_anxiety + trait_anger + stim_rating+ affordance + choice + affordance:choice + (affordance + choice + affordance:choice|subject) + (affordance + choice + affordance:choice|type)

fear_rating ~ trait_anxiety + trait_anger + stim_rating+ affordance + choice + affordance:choice + (affordance + choice + affordance:choice|subject) + (affordance + choice + affordance:choice|type)

"Fear_rating" and "anger_rating" are participants' emotional ratings during the task. "Trait_anxiety" and "trait_anger" are questionnaires response scores from the STAI inventory and Anger-approach inventory. "Affordance" and "choice" represent affordance conditions and choices made in a specific trial. And lastly, "type" represent the different movie clips used throughout the experiment

Supplementary Stimulus

A stimulus presentation example is shown below

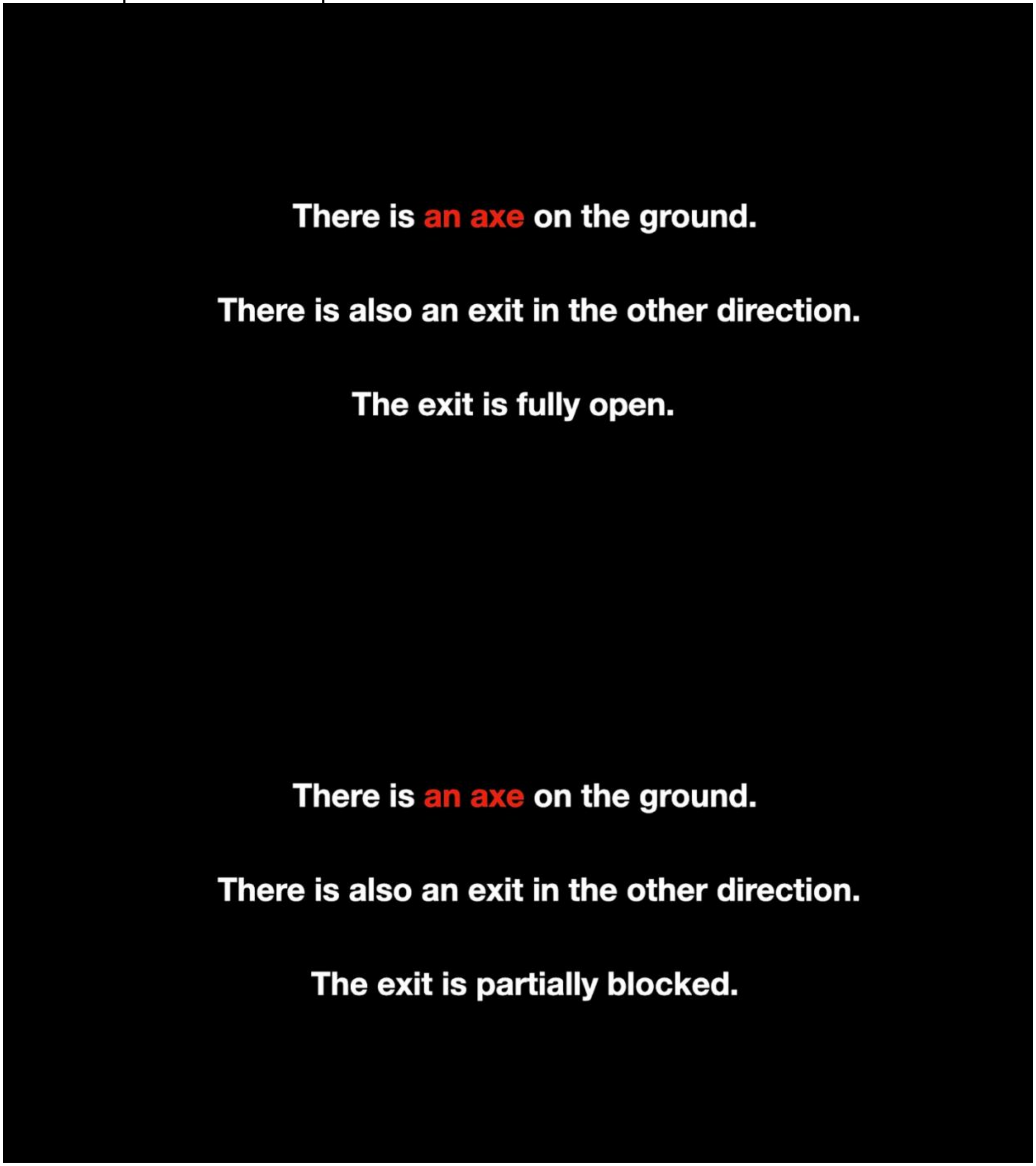

There is a mop on the ground.

There is also an exit in the other direction.

The exit is fully open.

There is a mop on the ground.

There is also an exit in the other direction.

The exit is partially blocked.

List of contingency items

Weapon-Conditions	Non-Weapon Conditions
Axe	Mop
Iron Rod	Wooden Cane
Rifle	Violin
Iron Hook	Heavy Book
Hammer	Heavy Suitcase
Barbell	Basketaball
Fire Extinguisher	Clay Pot
Long Shovel	Golf Stick
Large Wrench	Large Vase
Kitchen Knife	Paper Cutter

Escapeble-Conditions	Non-Escapeble Conditions
Open Window	Boarded Window
Open Door	Locked door
Tunnel	Wall
Exit	Deadend
Gate	Fence
Road	Blockade
Open Vent	Sealed Vent
Clear Hallway	Broken Floor

Supplementary Material – Pilot Study

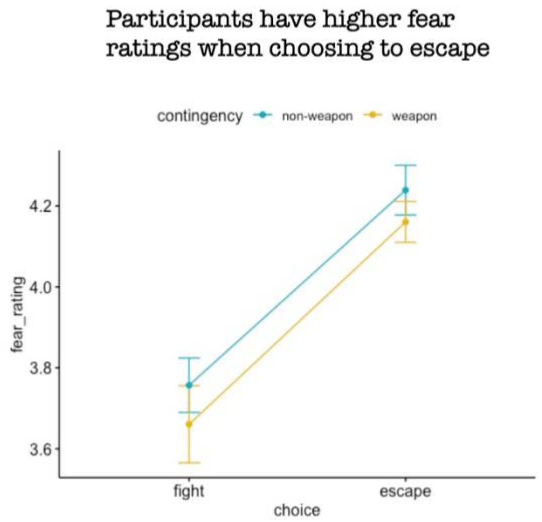

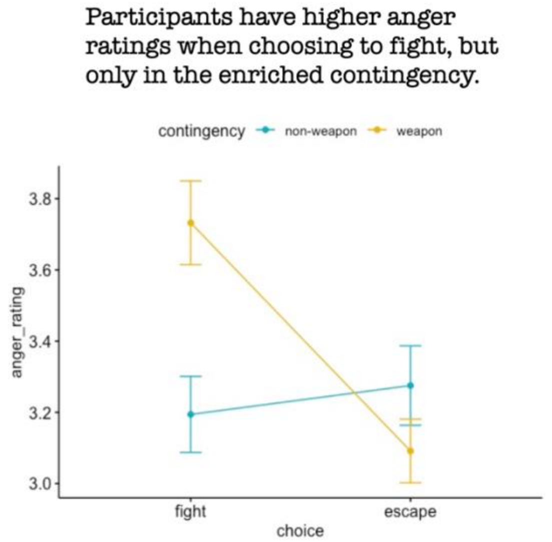

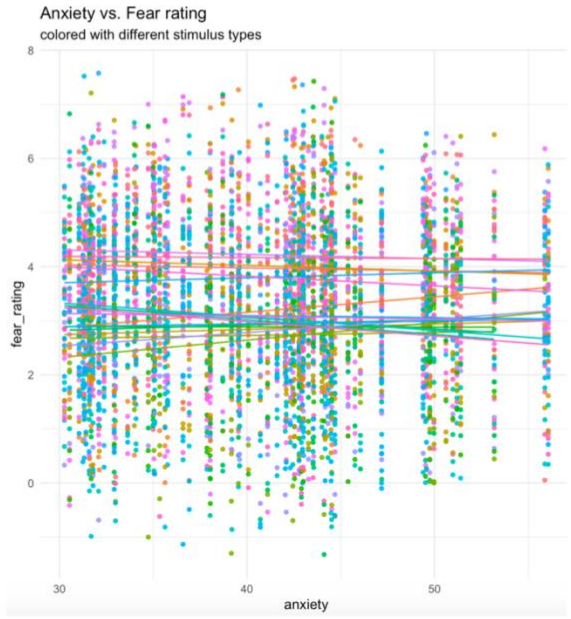

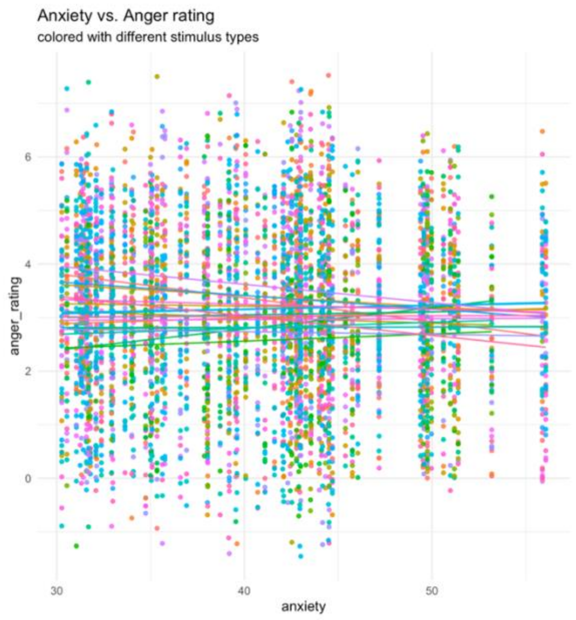

Movie clips plot synopsis

1. The protagonist finds himself inside an emergency tunnel flashing with red lights. He looks around with unease. Slowly, a figure emerges from afar, with its nature hidden in darkess. After a while, the figure reveals itself to be a frightening monster with dishaped skin. The protagonist runs away towards a door with fear.

2. Thr protagonist finds herself inside a room with dim lights. The lights are flickering, and she looks around with fear. After a while, a black monster figure suddenly appears above her on the ceiling, and prepare to attack her. She falls on the ground, but avoided and attack and plans for her next move.

3.The protagonist tiptoes through a decrepit factory. Suddenly, a creature made of steel and wires with red glowing eyes starts chasing him down the echoing halls. He ducks and weaves through old machinery to evade capture.

4.The protagonist steps carefully in an ancient crypt, but his touch awakens skeletal figures. They rise, clattering in the dim light. He turns and sprints, trying to find his way out through the twisting underground maze.

5.Enveloped in a dense fog, the protagonist hears a sorrowful wail. A ghostly figure appears, its face indistinct. She spies a faint light in the distance and makes a break for it, the ghost's cries echoing behind her.

6.The protagonist is alone on a deserted space station. A slimy alien with long tentacles glides silently in zero gravity. He holds his breath and moves without a sound, trying to outmaneuver the alien to reach a safe compartment.

7.In a dusty library, the protagonist finds an ancient, ornate book. As he reads, inky shadows begin to emerge, reaching towards him. He scrambles to shut the book and contain the darkness spilling out.

8.At a twisted carnival, the protagonist notices the ringmaster's sinister smile. She is the prey in his deadly game. She ducks behind tents and dodges booby traps, trying to outpace his cunning tricks.

9.As darkness descends, the protagonist meets beings with eyes that burn in the night. They call out, mimicking the voices of loved ones. He covers his ears and rushes away, trying not to be tricked by their calls.

10.Deep under the sea in a shipwreck, the protagonist is cornered by eerie creatures, part fish, part human. She sees a glimmer of light above and swims with all her might, the monsters close on her heels.

11.In a cursed town, the protagonist watches in horror as neighbors transform into hideous forms. He runs through local lore in the town square, seeking a way to lift the curse while avoiding the glares of the changed.

12.Pulled into a distorted dimension, the protagonist stands face-to-face with a being with a mirror for a face. Her worst fears reflected back at her, she stands her ground, looking for the courage to turn away. The mirror monster further approaches with a twisted smile.

13.In the pitch black, the protagonist feels the darkness tug at his feet. Lighting a match reveals creeping shadow tendrils. He lights more candles, trying to hold back the living darkness that thirsts for him.

14.Inside a grand mansion, the protagonist feels the eyes of painted figures following her. When she solves a hidden puzzle, the figures begin to step out of their frames, and she must escape before they fully emerge.

15.Walking through a ghost town, the protagonist encounters the specter of a lawman reliving his last gunfight. He tries to intervene, hoping to give the sheriff peace and release himself from this spectral loop.

16.Trapped in a blizzard, the protagonist finds a lonely lodge. As the temperature drops, an ethereal figure made of ice and snow slides through the walls. He throws logs on the fire, trying to keep the spectral chill at bay.

17.The protagonist stumbles into a garden where the statues seem to whisper in the wind. When she realizes they are the petrified remains of previous visitors, she runs from the sinister gardener who tends to this deadly flora.

18.Hiking through the forest, the protagonist is stalked by a shape-shifting spirit that blends with the trees and bushes. He pauses, trying to distinguish between the rustling leaves and the camouflaged predator.

19.Seeking shelter, the protagonist hides in an ornate theater. Ghostly applause fills the air as transparent patrons appear in the seats. She steps on stage, acting out a scene to appease the phantom audience and prevent her eternal encore.

20.In a dystopian city, the protagonist dashes through the streets, pursued by relentless, mechanical hounds. He ducks into alleys and behind debris, using his knowledge of the city's ruins, trying to outsmart their advanced tracking systems.

Supplementary Discussion

1. On the differential effect on fear and anger from environmental affordance

We do not have an a priori theory about such a differentiation between fear and anger, hence the following should be understood as post-hoc speculation of the phenomenon we observed. Agency, or the sense of control over one's actions and their outcomes, is a critical factor in the experience of anger. Access to a weapon in a threatening scenario might enhance the sense of agency, providing individuals with a means to potentially alter the outcome through their actions, thus potentially heightening the expression of anger. In contrast, fear may be more primal and automatic, requiring less reference to sense of agency for its response. Fear is triggered by the perception of imminent danger and activates a fast, subcortical pathway in the brain that bypasses the higher cognitive processing centers (LeDoux, 1996). This pathway enables rapid response to threats, such as fleeing, freezing, or hiding. The availability of an escape route offers a means to avoid the threat, but does not necessarily increase fear, contrarily it might reduce the uncontrollability and perceived vulnerability to the threat from which fear originates (Armfield 2006). The sense of control provided by the presence of an escape route might reduce the unpredictability associated with the threatening situation, which is a key element in the amplification of fear. Also, the ability to escape forges the subject's appraisal of the situation, from one that might be perceived as overwhelmingly threatening and fearful (no escape) to one that is manageable (escape available).

Also, while fear is a response to immediate threat, anger may require a more complex cognitive appraisal of the situation, including considerations of personal values, norms, and the specifics of the threat. This appraisal process can be influenced by the presence of a weapon, or affordance in general.

2. On the data quality and replication from online participants

Large sample and adequate power: we have recruited overall 650 (including pilot) participants, which by any standard in experimental psychology is very large and affords us the power to detect differences.

Stringent participant selection criteria: we have applied very rigorous criteria in recruiting subjects. Specifically, our participants have an average HIT (Human Intelligence Task) approval rate of 95% or higher and have completed a minimum of 1000 HITs. This ensures both the response quality and engagement from the online participants.

In terms of data quality from online studies: comparative studies have found that online recruitment can be at least as good if not superior to in person recruitment, with the aid of larger study population and stringent selection criteria (McRobert et al., 2018; Frampton et al., 2020)

Lastly, interms of Pre-registration, Data and Code availability: We ensure reproducibility by:

a. Piloting in a large enough sample to derive hypotheses.
b. Pre-registering these.
c. Conducting our main experiments in large samples.
d. Making available our data and code to the public alongside our article.

For these reasons we believe that our study is highly reproducible with validity.
